# Influence of patient characteristics on *Helicobacter pylori* eradication with Vonoprazan: A subgroup analysis of the pHalcon‐HP trial

**DOI:** 10.1002/jgh3.70044

**Published:** 2024-11-15

**Authors:** William D Chey, Francis Mégraud, Loren Laine, Neila Smith, Eckhard Leifke, Barbara Hunt, Colin W Howden

**Affiliations:** ^1^ Gastroenterology and Nutrition Sciences, Division of Gastroenterology and Hepatology, Department of Internal Medicine Michigan Medicine Ann Arbor Michigan USA; ^2^ Emeritus, INSERM U1312 University of Bordeaux Bordeaux France; ^3^ Medicine (Digestive Diseases) Yale School of Medicine New Haven Connecticut USA; ^4^ Attending Physician, VA Connecticut Healthcare System West Haven Connecticut USA; ^5^ Phathom Pharmaceuticals, Research and Development Buffalo Grove Illinois USA; ^6^ Emeritus, Department of Medicine University of Tennessee College of Medicine Memphis Tennessee USA

**Keywords:** clinical trial, *Helicobacter pylori*, potassium competitive acid blockers, proton pump inhibitors, vonoprazan

## Abstract

The efficacy of vonoprazan‐based dual and triple therapy vs. lansoprazole‐based triple therapy in the treatment of *H. pylori* infection was largely consistent regardless of age, sex, race, ethnicity, BMI, alcohol intake, smoking status, and study drug compliance.

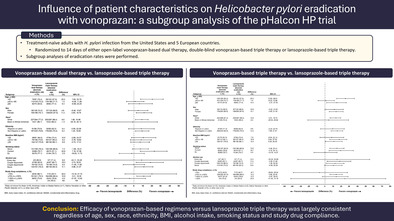

## Introduction

Half of the world's population is infected with *Helicobacter pylori* (*H. pylori*), with wide regional variation in prevalence.^1^ Approximately 80% of people with *H. pylori* infection are asymptomatic. However, all develop gastritis, 10% develop peptic ulcer disease,^2^ and a small percentage develop gastric adenocarcinoma or gastric marginal zone B‐cell lymphoma of mucosa‐associated lymphoid tissue.^2^

Since 1989, proton pump inhibitors (PPIs) have been considered part of *H. pylori* treatment regimens. By raising intragastric pH, they promote active replication of *H. pylori*, increasing the effectiveness of some antibiotics used to treat this infection.^2^ Currently, the triple combination of a PPI, clarithromycin, and amoxicillin (or metronidazole) remains one of the most commonly used regimens for the treatment of *H. pylori* infection.^3^ However, widespread use of macrolide antibiotics for other infections has led to an increase in the prevalence of *H. pylori* clarithromycin resistance, and a corresponding decline in eradication rates with clarithromycin‐based triple regimens.^4^ It has been recommended that clarithromycin should only be used in regions where resistance is known to be <15% and that its use be restricted to patients with no previous history of macrolide exposure for any reason.^4^

Vonoprazan, a potassium‐competitive acid blocker (P‐CAB), has been approved in Japan and several other countries for the treatment of *H. pylori* infection and other acid‐related diseases since 2014. In 2022, it was approved by the United States Food and Drug Administration (FDA) as a copackaged product with amoxicillin (dual regimen) or with clarithromycin and amoxicillin (triple regimen) for the treatment of *H. pylori* infection in adults.^5^ P‐CABs are pharmacokinetically and pharmacodynamically distinct from PPIs; they produce more rapid and profound inhibition of gastric acid secretion and may be dosed independently of mealtimes.^6^

The phase 3 pHalcon‐HP trial (NCT04167670) was conducted in treatment‐naive adults with *H. pylori* infection in the United States and five European countries.^7^ Patients (*N* = 1046) were randomized to 14 days of open‐label vonoprazan dual therapy, or double‐blind vonoprazan‐ or lansoprazole‐based triple therapy. Vonoprazan‐based dual and triple therapy were noninferior to lansoprazole‐based triple therapy for eradication rates in patients with nonresistant strains; both were superior to lansoprazole‐based triple therapy in patients with clarithromycin‐resistant strains, and among all patients, regardless of resistance status. The size of the pHalcon‐HP program created the opportunity to understand whether the magnitude of the treatment effect for vonoprazan versus lansoprazole varies across different subgroups.

To explore the possibility of differences in treatment effect among specific subsets of patients enrolled in pHalcon‐HP, we performed subgroup analyses of eradication rates among all patients, regardless of resistance status. Subgroups assessed included those based on baseline characteristics (age, sex, race, ethnicity, body mass index [BMI], self‐reported smoking, and alcohol use), and study drug compliance. Eradication rates were summarized by treatment group for each level in each subgroup. Within each level, the point estimate and two‐sided 95% confidence interval (CI) of the difference in eradication rates between each vonoprazan group and the lansoprazole group were calculated via the Miettinen and Nurminen method. For each subgroup, logistic regression was performed, including factors for treatment, subgroup, and treatment by subgroup interaction for the comparisons of vonoprazan dual and triple therapy with lansoprazole triple therapy.

To identify factors that may have influenced eradication rates, we performed stepwise logistic regression to fit two models. The first included subjects who received vonoprazan dual therapy or lansoprazole triple therapy. The second included subjects who received vonoprazan triple therapy or lansoprazole triple therapy. For both models, factors considered for inclusion included treatment group, clarithromycin resistance status, and the subgroup variables listed above. Statistical analyses were performed using SAS for Windows, version 9.4 (SAS Institute, Cary, NC).

The full analysis set used for this subanalysis included 992 patients (vonoprazan dual therapy, *n* = 324; vonoprazan triple therapy, *n* = 338; lansoprazole triple therapy; *n* = 330). Baseline characteristics were well balanced among the groups.

**Figure 1:**
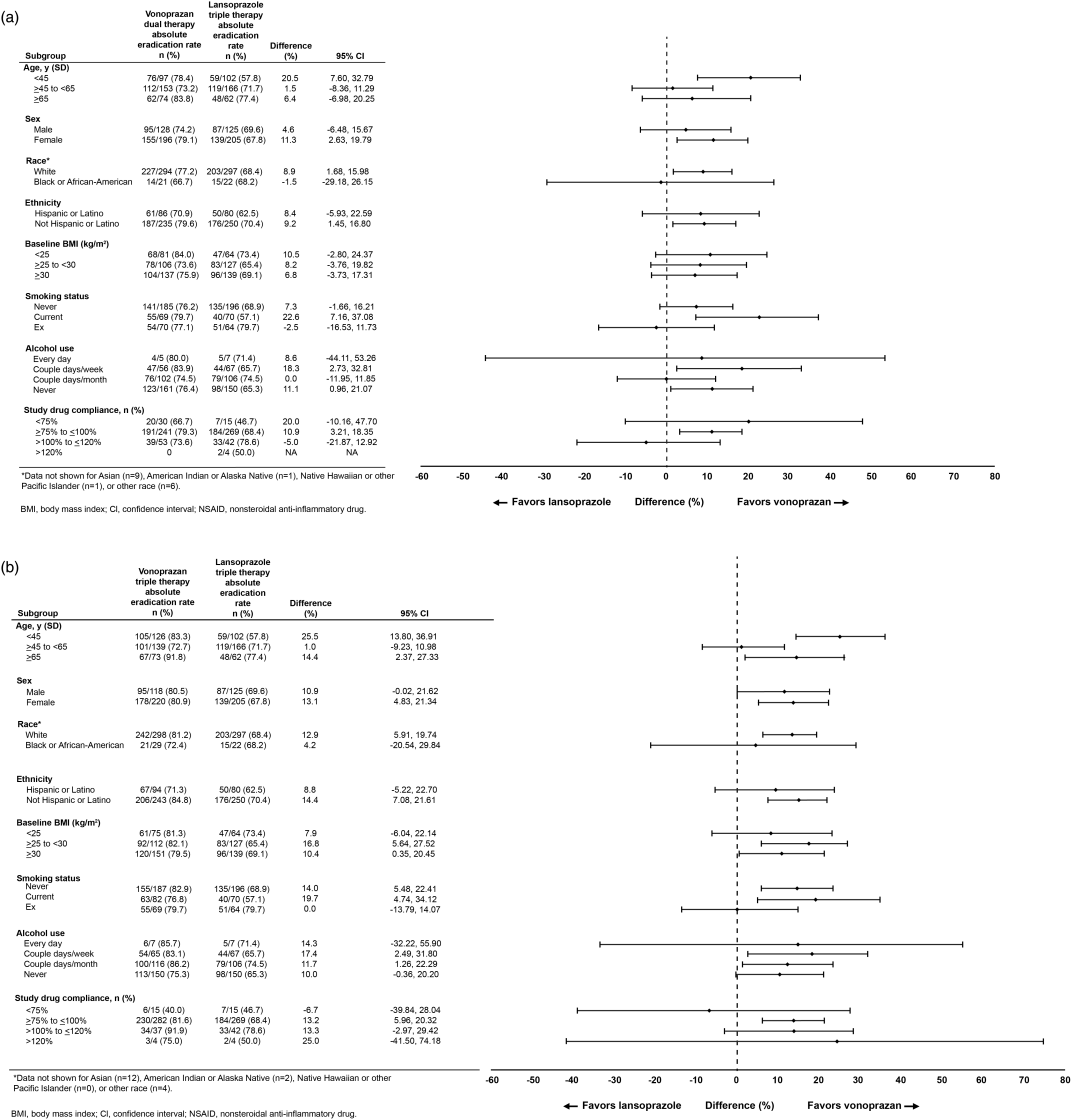
Differences in *Helicobacter pylori* eradication rates between (a) vonoprazan dual therapy and lansoprazole triple therapy or (b) vonoprazan triple therapy and lansoprazole triple therapy in all patients, according to baseline demographics and clinical characteristics.

Treatment group differences of vonoprazan dual and triple therapy compared with lansoprazole triple therapy across baseline demographic and clinical subgroups are shown in Figure 1a,b, respectively.

Multivariate modeling to explore the impact of each subgroup on eradication rates revealed that in both models, eradication rates were higher in subjects with *H. pylori* strains not resistant to clarithromycin (*P* < 0.0001 for both) and in older subjects (*P* = 0.0157 and *P* = 0.0193 for vonoprazan dual and triple therapy, respectively). In the vonoprazan triple therapy model, compliance (*P* = 0.0026) and alcohol use (*P* = 0.0410) were also significant factors; eradication rates were higher in subjects with better study drug compliance, and in subjects who used alcohol ≥2 days/month versus than those who never used alcohol.

## Discussion

pHalcon‐HP was the first trial to assess vonoprazan‐based dual and triple therapy in patients from the United States and Europe.^7^ In this *post hoc* analysis, the magnitude of the treatment effect for vonoprazan versus lansoprazole in *H. pylori* eradication was generally comparable, regardless of most demographic and clinical characteristics, consistent with the results in the overall group of patients. Of note, age group significantly influenced relative eradication rates, with the greatest difference seen in patients aged <45 years in favor of vonoprazan‐based therapies. Although we can only speculate on the reason for this difference, the phenomenon of reduced adherence to medications among younger patients has been demonstrated in multiple studies.^8^, ^9^ PPIs require acid activation and thus should ideally be administered ~30 min prior to a meal,^10^ whereas the relative timing of food intake does not influence vonoprazan pharmacodynamics.^4^ Younger patients may have been less likely to adhere to dosing recommendations for lansoprazole relative to meals.

As all patients in this study were treatment‐naive, results cannot be generalized to patients with persistent *H. pylori* infection despite previous treatment. Some subgroups had relatively small numbers of patients, resulting in correspondingly wide confidence intervals. This precludes any firm conclusions regarding relative efficacy of vonoprazan‐based regimens versus lansoprazole triple therapy in these subgroups. The potential marginal benefit of alcohol use on eradication rates with vonoprazan‐based triple therapy has no obvious explanation and could even represent a chance finding among the multiple subgroup analyses.

In conclusion, the relative efficacy of vonoprazan‐based regimens versus lansoprazole triple therapy was consistent regardless of age, sex, race, ethnicity, BMI, alcohol intake, smoking status, and study drug compliance.

## Ethical approval

The trial was approved by the institutional review boards of participating institutions, and written informed consent was obtained from participants before enrollment. The trial was conducted in accordance with the Declaration of Helsinki and the International Conference on Harmonization Guidelines for Good Clinical Practice.

## Data Availability

Individual participant data will not be shared.
